# Aetiology of Acute Undifferentiated Fever Among Children Under the Age of Five in Vietnam: A Prospective Study

**DOI:** 10.1007/s44197-023-00121-4

**Published:** 2023-05-31

**Authors:** Xuan Duong Tran, Van Thuan Hoang, Thi Thuy Duong Dang, Thi Phuong Vu, Minh Manh To, Trong Kiem Tran, Manh Dung Do, Duy Cuong Nguyen, Quoc Tien Nguyen, Philippe Colson, Philippe Parola, Pierre Marty, Philippe Gautret

**Affiliations:** 1 Thai Binh University of Medicine and Pharmacy, Thai Binh, Vietnam; 2 IHU-Méditerranée Infection, Marseille, France; 3 Aix Marseille Univ, IRD, AP-HM, SSA, VITROME, Marseille, France; 4 Thai Binh Paediatric Hospital, Thai Binh, Vietnam; 5 Aix Marseille Univ, IRD, AP-HM, MEPHI, Marseille, France; 6 Université Côte D’Azur, Inserm, C3M, Nice Cedex 3, France; 7 Parasitologie-Mycologie, Centre Hospitalier Universitaire L’Archet, Nice Cedex 3, France; 8 VITROME, Institut Hospitalo-Universitaire Méditerranée Infection, 19–21 Boulevard Jean Moulin, 13385 Marseille Cedex 05, France

**Keywords:** Fever without a source, Children, Enterovirus, Rash

## Abstract

**Background:**

To investigate the aetiology of acute undifferentiated fever (AUF) among children under the age of five in Vietnam.

**Methods:**

This prospective study was conducted in the Thai Binh paediatric hospital, between July 2020 and July 2021 among children with AUF at admission. Real-time PCR testing 18 microbial pathogens were done on blood samples.

**Results:**

286 children were included, with median age of 16 months. 64.7% were male. 53.9% were positive for at least one pathogen by PCR. Enterovirus, human herpesvirus 6, adenovirus, and varicella zoster virus PCR were positive for 31.1, 12.6, 1.4, and 1.0% patients, respectively. Other pathogens tested negative by PCR. During the hospital stay, based on clinical criteria 47.2% children secondarily presented with signs of respiratory tract infections, 18.9% had hand, foot and mouth disease, 4.6% had chickenpox. 4.2% presented signs of central nervous system infections, 1.0% had dengue (antigenic test) and 1.0% had signs of gastrointestinal infection. Finally, 23.1% patients presented a fever with or without a rash and no other symptoms and ultimately received a diagnosis of AUF.

**Conclusion:**

Real-time PCR of blood is useful for detecting pathogens and diagnosing infectious causes of AUF. Further prospective studies with blood and urine culture testing and PCR investigation of not only blood but also cerebrospinal fluid, throat, and skin samples according to symptoms would be of interest to confirm the predominance of viral infections in children with AUF and to guide therapeutic options.

**Supplementary Information:**

The online version contains supplementary material available at 10.1007/s44197-023-00121-4.

## Background

Fever is a common complaint in children. It is a sign of an underlying illness that warrants investigation, especially if the child shows warning signs or persistent fever. It is a significant clinical sign and the first step in controlling fever is to identify its cause. Once the cause is known, the primary reason for treating a fever is to improve the child’s comfort [1, 2].

Evaluating children with fever, especially those under 36 months of age, is a challenge for clinicians in the emergency department and for family physicians. Although guidelines have been developed to help with the triage and management of febrile children so that they can be safely monitored at home or hospitalised, no particular plan has proven to be completely satisfactory [3].

Undifferentiated fever or undifferentiated febrile illness is a common clinical problem, particularly in children. It is defined as fever with no known source of infection based on history, physical examination and/or rapid laboratory testing [4]. Undifferentiated fever might be referred to in the literature as acute undifferentiated fever (AUF) or fever of unknown origin. In the 1960s, fever of unknown origin was defined as a fever lasting at least 21 days with no apparent source after one week of investigation [5]. However, AUF was recently defined as a temporary febrile illness limited to three weeks accompanied by non-specific symptoms [6]. In tropical countries, AUF is sometimes referred to as “acute undifferentiated febrile illness”, “non-malarial fever”, “non-malaria fever” or even “acute fever of unknown origin” in several studies.

Managing patients with AUF is a challenge for physicians. In a systematic review of 18 studies, 51% children with AUF had an infection, 26% had a non-infectious condition, with collagen vascular disease and malignancy the most frequent, and 23% had no diagnosis [7]. Although a fever may clear up on its own in some cases, in others a serious bacterial infection can develop [8]. The response to antipyretics does not differentiate bacterial from viral infections [9]. In most cases, a febrile child has additional symptoms and signs of an acute infection.

Recently, methods of diagnosing the cause of fever have been developed, including microbiological analysis, easily accessible computed tomography (CT) and magnetic resonance imaging (MRI), and rapid genetic analysis for autoinflammatory diseases [10]. However, in low- and middle-income countries, as well as in medical facilities where there are not enough diagnostic facilities, finding the cause and providing care for children with fever is still difficult. Empirical treatment by clinicians, particularly with the widespread use of antibiotics, leads to inappropriate treatment and may increase the prevalence of antibiotic resistance.

Epidemiologcical data on the aetiology of fever helps guide clinicians with diagnosis and and choice of an appropriate treatment methods. Blood PCR testing plays a valuable role in the identification of infectious causes of acute undifferentiated fever in children. Indeed, PCR testing allows for the rapid and sensitive detection of genetic material from various pathogens, such as bacteria, viruses, and parasites, present in the blood. PCR testing can simultaneously screen for multiple pathogens, including those responsible for common febrile illnesses in children, such as dengue, malaria, typhoid fever, respiratory viruses, and bacterial infections. By identifying the specific pathogens in febrile patients, it facilitates early and accurate diagnosis, which is crucial for appropriate treatment and management. PCR testing aids in differentiating between various infectious causes, guiding clinicians toward appropriate treatment strategies. In fact, identifying the causative pathogen through PCR testing enables healthcare providers to make informed decisions regarding antimicrobial therapy, antiviral treatment, or supportive care based on the specific diagnosis. Therefore, we conducted this study on children under the age of five who presented with an unexplained fever, with or without skin symptoms, upon admission the at Thai Binh Paediatric Hospital, in Vietnam. A molecular epidemiological investigation was also conducted based on blood samples to evaluate the usefullness of PCR in identifying microbial pathogens as responsible for blood stream infection causing AUF among children.

## Materials and Methods

### Study Setting

The Thai Binh Paediatric Hospital (TBPH) is located in the Thai Binh Province, in the north of Vietnam, 110 km from the capital, Hanoi. In 2019, the city of Thai Binh had a population of 1,860,447 inhabitants, with 89.4% of people living in rural areas and 28.3% aged between 0 and 19 years old [11]. This hospital has 565 beds divided into 15 different medical wards, and employed 86 paediatricians, and 171 nurses in 2019. Patients present at TBPH either directly from their homes or when referred from district hospitals. Children can be transferred to the National Hospital of Paediatrics in Hanoi when the medical situation goes beyond the expertise available at TBPH [11, 12]. The average number of monthly hospitalisations is around 2,000 and is often more, especially during the peak of infections [12]. Microbiological investigations at TBPH are limited [12, 13].

### Study Site and Criteria for Selection

This prospective study was conducted in TBPH between July 2020 and July 2021. Patients under the age of five who were hospitalized with AUF were included from three wards: the intensive care unit (ICU), the infectious diseases ward, and the neonatology ward.

The criteria for inclusion in this study were: patients with an isolated fever (axillary temperature > 38.0 C) on admission or with fever associated with skin signs were included. Children over the age of five, as well as HIV seropositive, immunocompromised or neutropenic patients and those presenting with travel-related fever were excluded from this study.

### Clinical Data and Specimen Collection

Demographic, epidemiological and clinical data were collected using standardised questionnaires. According to the expanded programme on vaccination in Vietnam [14], the tuberculosis vaccine is recommended as soon as possible within 30 days of birth. Three doses of the pentavalent vaccine (diphtheria, tetanus, pertussis, hepatitis B, and *Haemophilus influenzae* type B) and oral polio vaccine are recommended at 2–4 months. The measles vaccine is recommended at 9–11 months. A lack of immunisation is defined as not receiving at least one of the recommended vaccines according to the Vietnamese programme.

Children were considered to be at high risk of serious infection if they were less than one month old or presented with pale, mottled skin, lethargy or drowsiness or grunting/tachypnoea for those aged one month and over [15].

Routine laboratory investigations were performed in the TBPH at the discretion of the receiving clinician and were based on hospital guidelines. Blood specimens were collected from all patients for white blood cell (WBC) counts and C-reactive protein (CRP) measurement. In patients with suspected sepsis, blood cultures were performed on a sample of 2–5 mL venous blood, inoculated into 20 mL culture medium [brain heart infusion broth (Oxoid, Basingstoke, UK) plus 0.05% sodium polyanethol sulfonate (Sigma-Aldrich, St. Louis, MO, USA)]. The vented bottles were incubated at 37 °C for up to seven days. Bottles were routinely sub-cultured onto solid media after 24 h and seven days, with additional subculture if turbidity was noted upon daily inspection. Bacterial isolates from blood were identified using VITEK Mass Spectrometry System (Vitek MS; bioMérieux, Durham, NC, USA). In addition, influenza A and B was identified using an antigen rapid test (ASAN Easy Test Influenza A/B, ASAN PHARMACEUTICAL CO., LTD, Korea) with nasopharyngeal samples. Children presenting ≤ five days after the onset of fever were tested for dengue virus using the Dengue NS1 Ag rapid test (Dengue NS1 Ag SD BIOLINE, Korea).

Two millilitres of blood was systematically collected from participants by nurses and transferred into an EDTA tube. After collection, samples were stored at -80 C within one hour, and were then transferred to the Institut Hospitalo-Universitaire, Méditerranée Infection, Marseille, France, on dry ice before processing.

### PCR Procedure

Because we aimed to investigate pathogens responsible for blood stream infection, hence only blood samples were collected for PCR testing. DNA and RNA were extracted from the blood samples using the Thermo Scientific KingFisher Flex with the Kit NucleoMag^®^ Dx Pathogen (MACHEREY-NAGEL, Germany). Pathogens based on DNA were tested by one-step simplex real-time quantitative RT-PCR amplification, performed using the LightCycler^®^ 480 Probes Master kit (Roche Diagnostics, France). In contrast, pathogens based on RNA were tested by one-step simplex real-time quantitative RT-PCR amplification, performed using Multiplex RNA Virus Master Kit (Roche Diagnostics, France). Extraction products from blood samples were tested for 18 pathogens by RT-PCR: *Coxiella burnetii (1111* gene*), Borrelia* sp. *(bor16S* gene*), Streptococcus pneumoniae (plyN* gene*), Streptococcus agalactiae (Cfb* gene*), Salmonella* sp. *(invA* gene*), Staphylococcus aureus (nucA* and *Amydo* genes*), Tropheryma whipplei (Twhip_2* gene*), Rickettsia* sp. *(1029* gene*), Rickettsia felis (bioB* gene*), Bartonella* sp. *(ITS* gene*), H. influenzae (SDH* gene*), Orientia tsutsugamusi (TSULC* gene*)*, adenovirus, enterovirus, human herpesvirus 6, dengue virus, parechovirus, and varicella-zoster virus (in-house RT-PCR). These pathogens were selected based on their prevalence in patients with AUF, as reported in previous studies [16–20].

Negative control (PCR mix) and positive control (DNA from bacterial strains or RNA from viral strains) were used in each run. The results were considered to be positive for bacteria or virus amplification when the cycle threshold (CT) value was ≤ 35. A threshold value of 35 was used in each test run and we calculated the cut-off value as recommended by the CFX Manager Software Version 3.1 (Bio-Rad) to verify positive cases. Results were considered positive when the cycle threshold value of real-time PCR was greater than the cut-off value.

### Data Analysis

Data were double entered using Microsoft Access and were then cleaned and exported to STATA software version 17.0 (Copyright 1985–2021 StataCorp 4905 Lakeway Drive College Station, Texas 77845, USA) for analysis. Continuous variables were analysed and expressed as median and range. Categorical variables were presented as numbers and proportions.

Our main outcome was positive for at least one pathogen. The main predictive factors associated with detecting a pathogen in the blood samples were demographic data, clinical symptoms, and routine blood testing results (WBC count and CRP). WBC counts were analysed according to the children’s reference ranges for routine haematology tests, published by the North Bristol NHS Trust [21]. Only variables with a prevalence ≥ 5.0% were introduced into the statistical analysis. Bivariate analysis was used to calculate the Odds Ratio (OR) for the association between detecting a pathogen in blood samples and independent variables. Multivariate analysis was then adjusted for all variables with *P* values < 0.2 in the univariate analysis. Multivariate analysis was performed using exact logistical regression. A predictive factor was statistically significantly associated (*P* values < 0.05) with each pathogen or group of pathogens.

### Ethical Approval

The protocol was approved by the Thai Binh University of Medicine and Pharmacy institutional review board (No. 498/HDDD, project “Molecular epidemiology of infectious diseases among children under five years in Thai Binh, Vietnam”). The study was performed according to the good clinical practices recommended by the Declaration of Helsinki and its amendments. All parents or legal guardians of participants provided their written informed consent.

## Results

### Sociodemographic Characteristic of the Studied Population

**Fig. 1:**
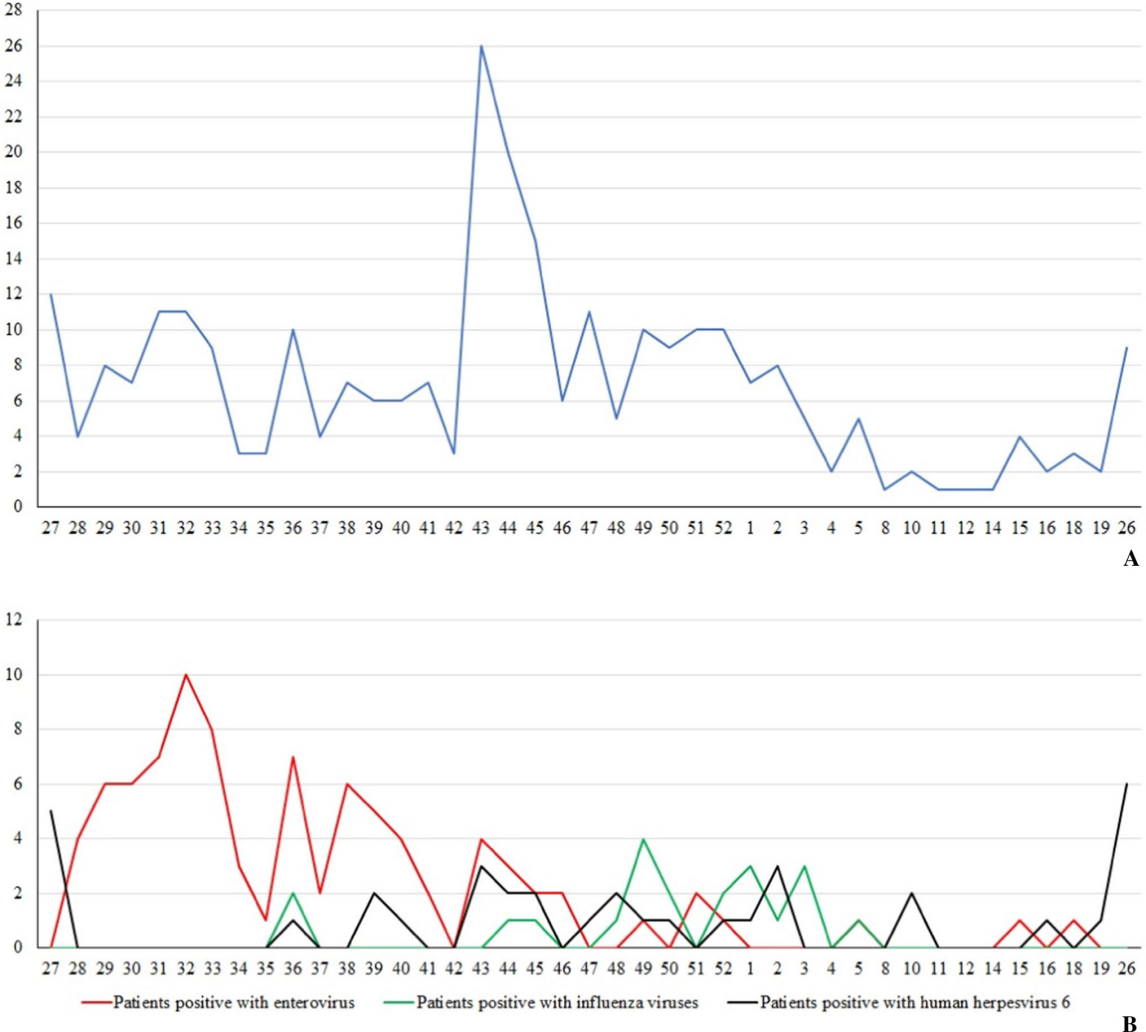
**A** Numbers of patients with fever by week (2020–2021). **B** Number of patients with positive PCR

A total of 286 children under the age of five were enrolled in this study throughout the year, with a peak in weeks 44–45 and a decrease in weeks 10–18 (Fig. 1). Of them, 185 (64.7%) were male. The median age was 16 months (ranging from 0 to 57 months) with 35 (12.2%) less than one month old and 243 (85.0%) patients less than 36 month olds. Regarding vaccination status, 261/286 (91.2%) children were fully vaccinated according to the expanded programme on vaccination in Vietnam.

### Clinical Features Upon Admission and Routine Laboratory Findings (Table 1)

**Table 1:** Clinical characteristics, routine laboratory findings and results of microbiological investigation performed at the Thai Binh Paediatric Hospital

	*N* = *286* *n (%)*
Clinical findings upon admission	
Skin symptoms	69 (24.1)
Rash	18 (6.3)
Blisters	48 (16.8)
Purpura	3 (1.0)
High risk of serious infection	39 (13.6)
Age < one month	35 (12.2)
Age ≥ one month	
Pale, mottled skin	0 (0)
Lethargy or drowsiness	2 (0.7)
Grunting or tachypnoea	2 (0.7)
WBC count	
Normal	102 (35.7)
Elevated	184 (64.3)
CRP (g/L)	
< 5	89 (31.1)
5– < 10	67 (23.4)
10– < 20	110 (38.5)
≥ 20	20 (7.0)
Blood culture (N = 41)	
*Haemophilus influenzae*	4 (9.8)
*Escherichia coli*	1 (2.4)
*Enterobacter* spp.	1 (2.4)
Influenza rapid test	
Influenza A positive	20 (7.0)
Influenza B virus positive	1 (0.3)
NS1 antigen rapid test^276a^	3 (1.1)

*WBC* white blood count, *CRP* C-reactive protein

^a^NS1 antigen rapid test was performed in 276 children with an onset of fever ≤ 5 days

A total of 53 (18.5%) patients were transferred from district hospitals or other medical centres. Fifty-five (19.2%) ill children had received antibiotics before admission, including 27 (9.4%) that were treated without medical prescription. The median time between the onset of symptoms and enrolment was one day, ranging from 0 to 14 days. Upon admission, 5.6% of children had adenopathy and 24.1% had skin symptoms including, notably, an erythematous rash and blisters.

Regarding the severity of disease, 39 (13.6%) children were at a high risk of serious infection, including 35 (12.2%) less than one month old and four children (1.4%) aged one month or over, with lethargy/drowsiness and grunting/tachypnoea.

A proportion of 64.3% children had an elevated WBC count and 45.5% had CRP levels ≥ 10 mg/L.

### Microbiological Findings at the TBPH Laboratory (Table 1)

Forty-one patients had a blood culture and six (14.6%) of these were positive, with *H. influenzae* being the most frequent. Only one patient was tested by urine culture and the result was negative.

All patients were tested using an influenza virus antigen rapid test. Twenty patients (7.0%) were positive for influenza A virus and one (0.3%) was positive for influenza B virus. A total of 276 patients with an onset of fever ≤ 5 days were tested for dengue virus using an NS1 rapid antigen test and three (1.1%) were positive. The weekly prevalence of influenza virus infections is presented in Fig. 1. Most cases were observed between week 44 and week 5.

### PCR Findings (Table 2)

**Table 2:** Blood pathogens identified by RT-PCR

Pathogens	N = 286 n (%)
**Enterovirus** ^a^	89 (31.1)
Human herpesvirus 6^a,b^	36 (12.6)
**Adenovirus**	4 (1.4)
Varicella zoster virus	3 (1.0)
At least one pathogen	131 (53.9)

No samples were positive for dengue virus, parechovirus, *Coxiella burnetii, Borrelia* sp*., Streptococcus pneumoniae, Streptococcus agalactiae, Salmonella* sp.*, Staphylococcus aureus, Tropheryma whipplei, Rickettsia* sp.*, Rickettsia felis, Bartonella* sp.*, Haemophilus influenzae* or *Orientia tsutsugamusi*

^a^One patient was co-infected with enterovirus and human herpesvirus 6

^b^One patient was co-infected with *Enterobacter* spp. (blood culture) and human herpesvirus 6

Of 286 patients, 131 (53.9%) were positive for at least one pathogen by PCR. Enterovirus, human herpesvirus 6, adenovirus, and varicella zoster virus PCR were positive for 89 (31.1%), 36 (12.6%), four (1.4%), and three (1.0%) patients, respectively. One case (0.4%) was positive for both enterovirus and human herpesvirus 6. Other pathogens tested negative by PCR. The weekly prevalence of enterovirus and herpesvirus 6 infections is presented in Fig. 1. Most enterovirus cases were observed between weeks 28–46, while no clear pattern was observed for herpesvirus 6 case distribution. There was a small peak in HHV-6 positive cases around weeks 26 to 27.

### Treatment and Outcomes of Patients (Tables 3)

**Table 3:** Final diagnosis, treatment provided at the TBPH and patient outcomes

	N = 286 n (%)
Final diagnosis	
Respiratory tract infections^a^	135 (47.2)
Hand, foot and mouth disease	54 (18.9)
Chickenpox	13 (4.6)
Central nervous system infections	12 (4.2)
Dengue	3 (1.0)
Gastrointestinal infections	3 (1.0)
Fever with or without rash, no other symptoms (fever without a source)	66 (23.1)
Empirical antimicrobial treatment	279 (97.6)
Cephalosporine	227 (79.4)
Beta-lactam	71 (24.8)
Aminoside	25 (8.7)
Carbapenem	2 (0.7)
Nitroimidazoles	1 (0.4)
Multiple antibiotics	64 (22.4)
Duration of antibiotic treatment (days), median (range)	4 (1–29)
Length of stay (days), median (range)	4 (1–29)
Outcomes	
Discharge	237 (82.9)
Discharge against medical advice	43 (15.0)
Transfer to national hospital	6 (2.1)
Death at hospital	0 (0)

^a^21 patients tested positive for influenza viruses

During their hospital stay, 135 children (47.2%) secondarily presented with signs of respiratory tract infections. A total of 54 (18.9%) had hand, foot and mouth disease and 13 (4.6%) had chickenpox, based on clinical criteria. Twelve children (4.2%) presented signs of central nervous system infections, three had dengue and three (1.0%) had signs of a gastrointestinal infection. Finally, 66 (23.1%) patients presented a fever with or without a rash and no other symptoms and received a diagnosis of a fever without a source.

A proportion of 97.6% of patients received antimicrobial treatment during their hospitalisation. Cephalosporin was the most common choice, followed by beta-lactam. It is notable that 22.4% patients received multiple antibiotics. The median duration of antibiotic treatment and hospitalisation was four days, ranging from 0 to 29 days. Among the 39 children at a high risk of serious infection, 36 received antibiotics, in line with treatment recommendations [15].

A proportion of 82.9% (237/286) of ill children were discharged and 15.0% (43/286) were discharged against medical advice. Six patients (2.1%) were transferred to the National Hospital for Paediatrics, No deaths were reported.

### Microbiological Diagnosis According to Syndromic Classification (Table 4 and Supplementary Fig. 1)

**Table 4:** Microbiological findings according to syndromic classification

	Respiratory tract infections N = 135 n (%)	Hand, foot and mouth disease N = 54 n (%)	Chickenpox N = 13 n (%)	Central nervous system infection N = 12 n (%)	Fever with purpura (N = 3) n (%)	Gastrointestinal infection, N = 3) n (%)	Fever with or without rash, no other symptom (acute undifferentiated fever) N = 66 n (%)
Rash	8 (5.9%)	7 (13.0)	1 (7.7)	1 (8.3)	0 (0)	0 (0)	1 (1.5)
Blisters	0 (0)	40 (74.1)	8 (61.5)	0 (0)	0 (0)	0 (0)	0 (0)
Purpura	0 (0)	0 (0)	0 (0)	0 (0)	3 (100)	0(0)	0(0)
Influenza rapid Ag test	21 (15.6)	0 (0)	0 (0)	0 (0)	0 (0)	0 (0)	0 (0)
Dengue rapid Ag test	0 (0)	0 (0)	0 (0)	0 (0)	3 (100)	0 (0)	0 (0)
Blood culture	4^1^ (3.0)	1^2^ (1.9)	1^2^ (7.7)	0 (0)	0 (0)	0 (0)	0 (0)
Total positive PCR	61^3^ (45.2)	24 (44.4)	4 (30.8)	4 (33.3)	0 (0)	2 (66.7)	36 (54.5)
Enterovirus	34 (25.2)	22 (40.7)	1 (7.7)	3 (25.0)	0 (0)	1 (33.3)	28 (42.4)
Human herpesvirus 6	27 (20.0)	1 (1.9)	1 (7.7)	1 (8.3)	0 (0)	1 (33.3)	5 (7.6)
Adenovirus	0 (0)	1 (1.9)	0 (0)	0 (0)	0 (0)	0 (0)	3 (4.5)
Varicella zoster virus	1 (0.7)	0 (0)	2 (15.4)	0 (0)	0 (0)	0 (0)	0 (0)
Total positive tests	80^4^ (59.3)	25 (46.3)	5 (38.5)	4 (33.3)	3 (100)	2 (66.7)	36 (54.5)

^a^Two patients were positive for *Haemophilus influenzae*, one patient was positive for *Enterobacter* spp. and one was positive for *Escherichia coli*

^b^One patient was positive for *Haemophilus influenzae*

^c^One patient was co-infected with enterovirus and human herpesvirus 6

^d^One patient was positive for HHV6 and *Enterobacter* spp., two were positive for HHV6 and influenza, and three were positive for EV and influenza

In total, a viral infection was identified in 45.5% of patients and a bacterial infection in 2.1% of patients. Among patients with symptoms of a respiratory tract infection, 59.3% had a positive test, with most frequent positive pathogens being influenza virus, enterovirus and human herpesvirus 6, and four had a positive blood culture. Among those with symptoms of hand, foot and mouth disease, 46.3% had a positive test with the vast majority being positive for enterovirus and one with a positive blood culture. Among the 13 patients with chickenpox symptoms, five were positive for a virus, including two for varicella zoster virus, and one had a positive blood culture. Among the 12 patients with central nervous system infection symptoms, four were positive for a virus (mostly enterovirus). The three patients with purpura were positive for dengue. Among the three patients with a gastrointestinal infection, two were positive for a virus. Finally, among patients with fever without a source, 54.5% had a positive test with the most frequent positive pathogen being enterovirus.

## Discussion

Fever in children is a common concern among parents and is one of the most frequent reasons for visiting the emergency department, often involving non-paediatric emergency physicians. The clinical assessment of fever in children is also markedly different from that of adult patients. Although the incidence of serious infections has decreased following the use of conjugate vaccines, fever remains a major cause of patient laboratory investigation and hospitalisation [22]. Most children with a fever and no focus of infection have a self-limiting viral illness that requires no treatment and disappears without sequelae [22]. In the majority of cases, the fever resolves on its own before diagnosis or develops further symptoms, leading to a diagnosis within the first seven days. A few however, may be at risk of developing a life-threatening infection [22]. Assessing the actual medical condition is paramount in order to accurately assess the severity of the disease and take appropriate and timely treatment. Physical examination, laboratory markers and microbiological investigation can help identify children at risk of serious infections and determine the appropriate antibiotic use in paediatric patients. In the absence of laboratory facilities, unexplained fever is a challenge for clinicians in deciding whether to administer antibiotics or to hospitalise patients.

Blood PCR testing offers significant advantages in the diagnosis of acute undifferentiated fever in children. Its rapid and sensitive detection, comprehensive pathogen coverage, specific diagnosis, tailored treatment approach, differentiation of similar clinical presentations, public health implications, and research utility make it an invaluable tool for healthcare providers in managing febrile illnesses and improving patient outcomes. This is significant in clinical practice because acute undifferentiated fever poses a diagnostic challenge as it can be caused by many infectious agents. Blood PCR testing provides a comprehensive approach by simultaneously screening for multiple pathogens, including bacteria, viruses, and parasites. This multiplexing capability enables the detection of many different pathogens in a single test, saving time and resources and improving diagnostic accuracy. In our study, 23% children received a final diagnosis of AUF, while the rest presented further symptoms during hospitalisation, leading to a specific diagnosis or a focused infection. Fifty-five percent of these patients with AUF were detected with at least one pathogen. In a systematic review addressing the causes of undifferentiated fever in children in South and Southeast Asia, a pathogen was identified in only 31% of cases [22]. The higher diagnostic rate in our study is likely to be due to the large panel of pathogens included in our protocol.

In our study, 15% of patients who underwent blood culture had a bacteraemia, with *H. influenzae* being the most frequently responsible pathogen. We tested blood for more than ten common bacterial pathogens using real-time PCR with no positive results, including for *H. influenzae*. This is likely to be explained by the lower sensitivity of the PCR method for diagnosing bacteraemia as compared to blood cultures, where much larger volumes of blood are processed. In the Wangdi review, given that fewer than 15% of children had blood culture and fewer than 1% had urine cultures, the bacterial causes of infection may have been underestimated in our survey.

We found that a high proportion of patients overall had a viral infection (46%), including those with AUF (54%). Wangdi et al*.* showed that viral infection was also the predominant cause of AUF among children in South and Southeast Asia, although a lower pooled positivity rate was reported (24%) [23]. The high rate of virus detection in Vietnamese participants corroborates finding in Swiss and US children [24, 25]. In one study conducted in a hospital in Switzerland, 35% of children presenting with AUF were positive for at least one virus including, notably, enterovirus (14%), herpesvirus 6 (11%), parechovirus (6%), and adenovirus (5%) [24]. In another study conducted in Missouri, United States, 62% of children with AUF were positive for at least one virus, including adenovirus (22%), herpesvirus 6 (17%), enterovirus (16%) and cytomegalovirus (6%) [25]. Our study shows that 31% of all participants were positive for enterovirus in the blood. This is likely to underestimate the true burden of enterovirus in this study, since respiratory, skin, cerebrospinal fluid (CSF) and stool samples were not collected in patients with relevant symptoms. In a previous study conducted among children in Thai Binh hospitalised for central nervous system infections, enterovirus was found in the CSF in 67% of cases [26]. Interestingly, the highest rates (41–42%) were observed in patients with hand, foot and mouth disease and AUF. In line with our finding, Lafolie et al*.* also showed that 35.2% of French children under the age of two, presenting with fever without source, sepsis, or suspected meningitis, were positive for enterovirus in the blood [27], and de Jong showed that 37% of Dutch children presenting sepsis-like syndromes were positive for enterovirus in the blood and/or the CSF [28]. Herpesvirus 6 was the second most common pathogen in our cohort (13%), with 8% among patients with AUF, a result in line with other studies [24, 25]. Higher rates (33%) of herpesvirus 6 infections were observed among febrile hospitalised children in an Iranian study [29]. Herpesvirus 6-related infections may be over-diagnosed, because the complete herpesvirus 6 genome can be integrated into the telomere of the host cell chromosome [30]. Such a situation may be suspected when herpesvirus 6 levels in whole blood exceed 5.5log_10_ copies/ml, which corresponds approximately to a CT < 20 in our experiments. However, all samples which were positive for herpesvirus 6 in our study had a relatively low viral load with a CT ≥ 29 (data not shown), suggesting that these were true infections.

Only a few cases of varicella were confirmed by blood PCR in our study, which is not surprising since skin samples are better suited for identification of this pathogen than blood samples, which are frequently positive in patients with acute zoster [31]. Unlike in other studies conducted in Asia where dengue was the commonest cause of AUF among children [23], we found only three cases of dengue through antigenic testing, none of which was confirmed by PCR and no cases of leptospira infection. Thai Binh is not in the endemic area of dengue fever in Vietnam, which explains the scarcity of cases in our study [32]. In a study conducted in southern Vietnam at community health posts and one clinic, 33.6% of patients presenting with acute undifferentiated fever were found to have dengue virus and acute primary infections were more common among children under the age of 15 than among adults [33]. It is notable that influenza was a common source of fever in patients who subsequently developed respiratory symptoms.

Regarding the treatment, 98% of children in our study were treated with an antibiotic, while 2% of them presented evidence of a bacterial infection. Indeed, in a five-year descriptive study, we showed that infectious diseases accounted for 61.0% of all hospitalised patients, while 81.4% of all cases received at least one antibiotic [12]. Guidelines are consistent in recommending systematic antibiotic treatment for children under the age of one month, however for children over the age of one month, most recommend antibiotic treatment only for those with a high risk of serious infection [15]. Our results suggest an overuse of antibiotics.

Our study has several limitations. Only inpatients were surveyed and the result might therefore not be representative of all paediatric populations with AUF over this period. It was conducted in a single provincial hospital, and the result might not represent other settings in Vietnam. The study was conducted during the second, third, and fourth waves of COVID-19 in Vietnam, with lockdown and restriction measures potentially complicating the inclusion of patients and affecting the epidemiology of pathogens responsible for AUF among children. On the other hand, we only investigated pathogens in blood samples, not on other specimens such as respiratory swabs and urine. On the other hand, the blood PCR test in this study only investigated 18 pathogens, so we could have missed some pathogens that were not included in the panel. We also did not type adenoviruses and enteroviruses although the latter primers we used did not amplify rhinovirus. Moreover, SARS-CoV-2 could also be a cause of AUF [34], although this was not tested in our study. Nevertheless, our study was conducted between July 2020 and July 2021, when very few COVID-19 cases were recorded in Vietnam [35]. Furthermore, we also did not perform serological testing, and AUF caused by some bacteria such as *Rickettsia* spp., *Leptospira* spp. might therefore have been underestimated due to the study design. Indeed, a previous study in Vietnam showed that only 56.0% of *R. typhi* infections identified through serological testing were positive for bacteria by qPCR testing [36]. Finally, our study did not include a control group an afebrile children, so we cannot be sure that the agents detected are the cause of AUF in children [36].

## Conclusion

The interest of PCR testing of blood, notably for enterovirus and herpesvirus 6 infections in children with AUF is confirmed by this study. Further prospective studies are recommended, involving systematic blood and urine culture in children under the age of one month [15]. PCR investigation of not only blood but also CSF, throat and skin samples according to symptoms would be of interest to confirm the predominance of viral infections in children with AUF and to guide therapeutic options.

## Supplementary Information

Below is the link to the electronic supplementary material.

Supplementary file1 (DOCX 97 KB)

## Data Availability

The data presented in this study are available on request from the corresponding author [VTH or PG] upon reasonable request.

## Abbreviations

AUF: Acute undifferentiated fever
CSF: Cerebrospinal fluid
TBPH: Thai Binh Peadiatric Hospital
