# Supplementary material for: Aetiology of Acute Undifferentiated Fever Among Children Under the Age of Five in Vietnam: A Prospective Study
Source: J Epidemiol Glob Health. 2023 May 31;13(2):163–72. doi: 10.1007/s44197-023-00121-4 (PMC10231849; doi:10.1007/s44197-023-00121-4)
Supplement: Supplementary file 1 — Supplementary file1 (DOCX 97 KB) [file 44197_2023_121_MOESM1_ESM.docx]

Supplementary Figure 1: Clinical signs at admission, diagnosis at discharge based on clinical status at admission and/or at follow-up and on results of diagnostic tests locally available, and positive pathogens detected by real-time PCR in the blood among 286 included patients


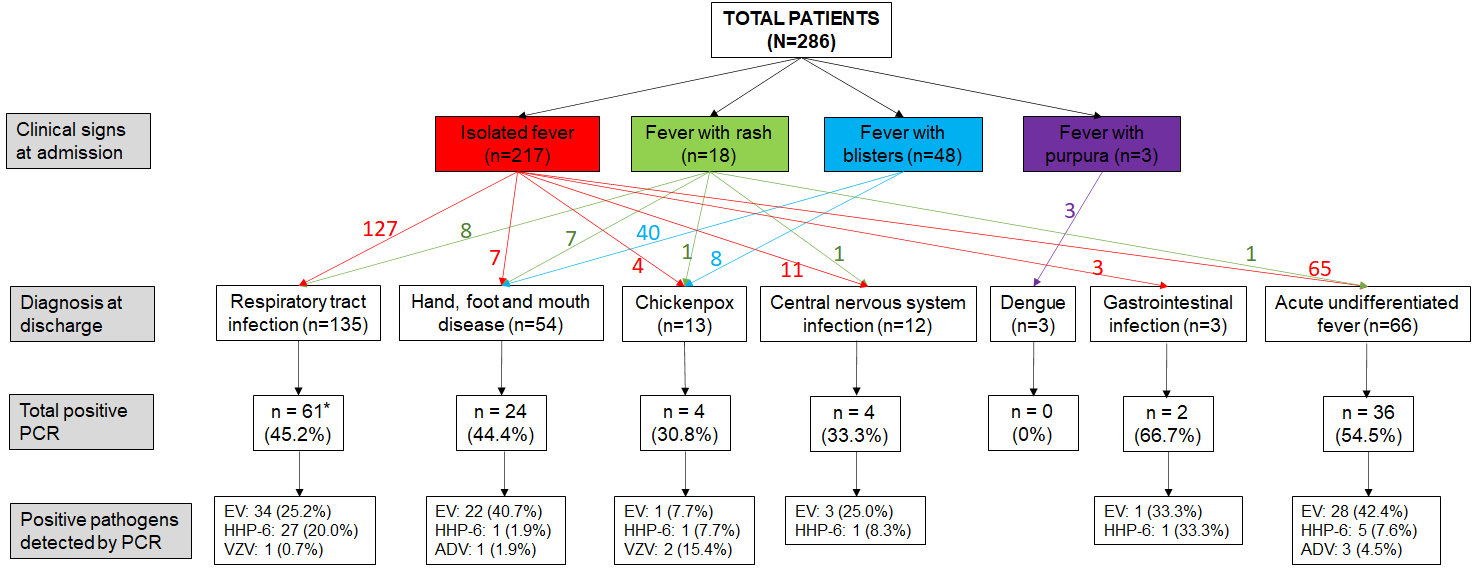


* One patient was co-infected with enterovirus and human herpesvirus 6; HHP-6: human herpesvirus 6; EV: enterovirus; VZV: varicella zoster virus
